# Reinforcement Learning for Bit-Flipping Decoding of Polar Codes

**DOI:** 10.3390/e23020171

**Published:** 2021-01-30

**Authors:** Xiumin Wang, Jinlong He, Jun Li, Liang Shan

**Affiliations:** 1 Key Laboratory of Electromagnetic Wave Information Technology and Metrology of Zhejiang Province, College of Information Engineering, China Jiliang University, Hangzhou 310018, China; 05a0303091@cjlu.edu.cn (X.W.); S1803081215@cjlu.edu.cn (J.H.); lshan@cjlu.edu.cn (L.S.); 2 Binjiang College, Nanjing University of Information Science and Technology, Wuxi 214105, China

**Keywords:** polar codes, reinforcement learning, successive cancellation, bit-flipping decoding, Q-learning-assisted decoding

## Abstract

A traditional successive cancellation (SC) decoding algorithm produces error propagation in the decoding process. In order to improve the SC decoding performance, it is important to solve the error propagation. In this paper, we propose a new algorithm combining reinforcement learning and SC flip (SCF) decoding of polar codes, which is called a Q-learning-assisted SCF (QLSCF) decoding algorithm. The proposed QLSCF decoding algorithm uses reinforcement learning technology to select candidate bits for the SC flipping decoding. We establish a reinforcement learning model for selecting candidate bits, and the agent selects candidate bits to decode the information sequence. In our scheme, the decoding delay caused by the metric ordering can be removed during the decoding process. Simulation results demonstrate that the decoding delay of the proposed algorithm is reduced compared with the SCF decoding algorithm, based on critical set without loss of performance.

## 1. Introduction

At present, the fifth generation (5G) mobile wireless communication system has begun to be commercially used. In 5G mobile wireless communication, polar codes have been used to transmit signal or synchronize data in control channel. In [1], Arıkan proposed the definition of polar codes and proved that it can reach the Shannon limit. When the code length goes to infinity, the channels are divided into noisy subchannels and noiseless subchannels, which are known as “channel polarization”. Arıkan also proposed a successive cancellation (SC) algorithm for polar codes, which uses the previously decoded bits as reliable information to assist in decoding subsequent bits. As the code length increases, the decoding performance of the SC algorithm gradually becomes better. However, for short or moderate code length of polar codes, the decoding performance of the SC decoding algorithm deteriorates due to the incomplete channel polarization. In order to improve the performance of the SC decoding algorithm, a successive cancellation list (SCL) decoding algorithm was proposed, which can greatly improve the decoding performance of polar codes [2]. The CRC-aided SCL (CASCL) algorithm uses the cyclic redundancy check (CRC) to improve the performance of the SCL decoding algorithm [3].

However, the complexity of the SCL algorithm is high. To reduce the complexity of the SCL algorithm, Orion et al. proposed the successive cancellation flip (SCF) decoding algorithm, which is a combination of the bit-flipping decoding and the SC decoding [4]. The SCF decoding algorithm needs low storage, which is the same as the storage of the SC decoding algorithm. The SCF decoding algorithm uses log likelihood ratio (LLR) as a metric to determine the flipping position. Chandesris et al. proposed an optimized metric which can improve ability of finding the first error bit in the initial SC decoding for the SCF decoding algorithm [5]. In order to further improve the performance of the SCF algorithm, they also proposed a dynamic SCF (D-SCF) decoding algorithm; it can dynamically build a list of candidate bits, which can guarantee that the next decoding attempt has the highest probability to correct the decoding error [6].

To further reduce the decoding complexity, Zhang et al. proposed a critical set, which has a high probability to include the first error bit in the initial SC decoding algorithm based on search tree [7]. Condo et al. proposed a fixed index selection (FIS) scheme and an enhanced index selection (EIS) criterion to reduce the decoding complexity of the SCF algorithm, which is based on the error distributions [8]. In [9], a path-metric-assisted SCF decoding algorithm was proposed, which can prioritize the bits that need to be corrected. Condo et al. proposed an improved SC-Flip multi-error decoding algorithm based on error correlation, which has better decoding performance than the SCF algorithm under the same decoding complexity [10].

However, the SCF decoding algorithm has a high complexity at low signal-to-noise ratio (SNR). To overcome this shortcoming, Lv et al. in [11] proposed a modified successive cancellation flip (MSCF) decoding algorithm, which can avoid the unnecessary flipping attempts of the SCF algorithm by using a threshold based on Gaussian approximation. Ercan et al. proposed a threshold SCF (TSCF) decoding algorithm, which uses a comparator to replace the LLR selection and ordering of the SCF algorithm to reduce the complexity of implementation [12]. Zhang et al. proposed an SCF decoder based on bit error rate evaluation, which can accurately locate the first error bit and correct it [13]. In addition, some authors in [14,15,16,17] used the segmented SC decoding algorithm to improve the error correction ability of the SCF decoding algorithm. Some characteristics of polar codes are used to segment the decoding process in these methods. For each segment, the decoding is performed by using a bit-flipping decoding algorithm.

Recently, machine learning (ML) techniques have been widely applied to many industries and research domains. In communication systems, ML-assisted communication systems have been widely studied.

For a deep learning technique, some authors in [18,19,20,21,22] used deep learning algorithms to assist in the coding and decoding of polar codes. To enhance the coding of the polar codes, a polar code construction algorithm based on deep learning was proposed [18], which has better performance than 5G polar codes without CRC [19]. Its core idea is that the bits of polar codes are regarded as the trainable weights of the neural network. In terms of the decoding algorithm, Gruber et al. achieved maximum a posteriori (MAP) bit error rate (BER) performance of polar codes by using a deep neural network to decode the codeword [20]. However, as the number of information bits increases, the complexity of learning also exponentially increases. In contrast, a neural SC (NSC) decoder based on the portioning technique decreases the complexity by learning information bits in each subblock of polar codes, and it reduces the decoding delay of the SC algorithm [21]. In order to improve the performance of the SC algorithm, a weighted successive cancellation (WSC) algorithm was proposed [22], which uses a neural network to learn the weights.

For reinforcement learning, some authors in [23,24,25] used reinforcement learning algorithms to assist in the coding and decoding of channel codes. In [23], a deep reinforcement learning method for constructing low-density parity-check (LDPC) codes was proposed, which combines a deep reinforcement learning training and a Monte Carlo tree search (MCTS). It has the potential to improve performance compared with traditional LDPC code construction. For further optimizing the structure of error correction codes (ECC), Huang et al. studied an artificial intelligence (AI)-driven method to design the ECC [24]. They also proposed a construction-evaluation framework, in which the construction framework can be implemented by various AI algorithms, such as reinforcement learning and genetic algorithm, and the evaluation framework can provide the performance metrics of the ECC. In [25], reinforcement learning-assisted bit flipping decoding was proposed. The authors studied the effective decoding strategy of Reed–Muller and BCH codes by using reinforcement learning methods. Traditional decoding algorithms are mapped to the Markov decision process (MDP), thereby allowing the optimal decision strategies to be obtained through training.

For the SCF decoding algorithm, it is critical to accurately locate candidate flipping bits and reduce decoding delay. In order to accurately locate the candidate bits, Wang et al. proposed a deep learning-assisted SCF decoding algorithm, using a long short-term memory (LSTM) network and reinforcement learning to find the error bits [26]. 

At present, little work has been devoted to applications of reinforcement learning on the SCF decoding of polar codes. The reinforcement learning algorithm can be used in the SC bit-flipping decoding, because the selection of candidate flipping bits can be seen as the decision process. The selection process of candidate bits is modeled as MDP. Compared with the traditional method, the reinforcement learning algorithm can quickly obtain the appropriate strategy. Therefore, the reinforcement learning is used to optimize the SCF decoding of polar codes in our design.

In this paper, we propose a Q-learning-assisted SCF (QLSCF) algorithm which uses the reinforcement learning to reduce decoding delay of the SCF decoding. First, we provide a set of candidate bits as the action space for reinforcement learning, and verify the completeness of the action space. Secondly, we design a one-bit SCF decoding algorithm assisted by reinforcement learning. Finally, the performance of the proposed QLSCF algorithm is compared with that of the traditional decoding algorithms, and the results are discussed. The structure of the paper is as follows. Section 2 introduces polar codes and reinforcement learning. The reinforcement learning model and the QLSCF decoding algorithm are shown in Section 3, and Section 4 discusses the simulation result and analysis. The conclusions are shown in Section 5.

## 2. Preliminaries

### 2.1. Polar Codes and Successive Cancellation Decoding Algorithm

Polar codes [1] are characterized by the two-tuple (N,K), where N denotes the code length, $N=2^{n}$, and K is the number of the information bits. The set including indexes of K information bits can be denoted as I, where I⊂(1, 2, 3,…,N). The remaining N−K bits are frozen bits, which are usually set to “0”. $u_{1}^{N}$ denotes the information sequence before encoding, and the encoding sequence $x_{1}^{N}$ can be obtained by Equation (1).

(1) $$x_{1}^{N}=u_{1}^{N}G_{N},$$

where $G_{N}$ is the generator matrix of polar codes [1]. $y_{1}^{N}$ is defined as the received data sequence, which is also the input data of the decoder.

The output data of decoder is denoted as ${\hat{u}}_{1}^{N}$, and the value of the LLR can be defined as Equation (2).

(2) $$L_{i}=ln\left(\frac{\mathrm{Pr}\left({\hat{u}}_{i}=0|y_{1}^{N},{\hat{u}}_{1}^{i-1}\right)}{\mathrm{Pr}\left({\hat{u}}_{i}=1|y_{1}^{N},{\hat{u}}_{1}^{i-1}\right)}\right),$$

The hard decision function can be defined as Equation (3).

(3) $${\hat{u}}_{1}^{i}=h\left(L_{i}\right)=\{\begin{array}{c} 0\;\;\;\;\;\;\;\;\;\;\;\;\;\;\;,i\notin I\\ \frac{1-sign\left(L_{i}\right)}{2}\;,\;i\in I \end{array},$$

To improve the decoding performance of the SC algorithm, the SCL algorithm and the SCF algorithm were provided [2,4]. The SCL decoding algorithm performs decoding by retaining l optimal candidate paths [2]. The SCL algorithm decodes a bit by splitting l paths into 2l paths and retaining l optimal candidate paths. After all of the bits have been decoded, the SCL algorithm selects the best path from l candidate paths as the decoded codeword. The CASCL algorithm uses the CRC check to improve the performance of the SCL decoding algorithm [3].

The SCF decoding algorithm contains a standard SC decoding and several additional flipping decoding attempts [4]. The SCF decoding algorithm needs to use the CRC check to judge whether the decoded codeword is correct. In the standard CRC scheme, CRC is appended to the tail of information bits. The specific process of the SCF decoding is as follows.

First, the SCF decoder performs a standard SC decoding to obtain ${\hat{u}}_{1}^{N}$, and ${\hat{u}}_{1}^{N}$ is verified by the CRC check. If it passes the CRC check, the SCF decoding outputs ${\hat{u}}_{1}^{N}$ obtained by the standard SC decoding. If it fails the CRC check, the SCF decoding goes to the bit-flipping decoding. The selection of the candidate bits is performed by comparing absolute value |L| of the LLR, and the bit with index of the smallest |L| is flipped firstly. In each bit-flipping decoding attempt, the one-bit SCF decoding is to flip only one bit in the decoded codeword of the initial SC. Then, the result of bit-flipping decoding is verified by the CRC check until the decoded codeword passes the CRC check. For the SCF decoding process, the effectiveness of the SCF decoder directly depends on the determination method of the candidate bits set.

For the SCF algorithm based on the critical set (SCF-CS), the structure of the critical set is very important. First, the algorithm establishes the binary tree corresponding to polar codes. Secondly, the algorithm decomposes the binary tree into several subblocks whose code rate is 1. Finally, the algorithm adds the first bit of these subblocks to the critical set. The SCF-CS decoding algorithm can effectively reduce the decoding complexity compared with the SCF algorithm [7]. Although the SCF-CS algorithm reduces decoding delay, it still has higher decoding delay compared with the SC algorithm at low SNR. In order to reduce the decoding delay, a new algorithm is proposed which uses the reinforcement learning to reduce the decoding delay of the SCF decoding.

### 2.2. Reinforcement Learning

Reinforcement learning is used to solve the problems of how to make agents use learning strategies to maximize rewards or achieve specific goals during the interaction with the environment [27]. The common model of reinforcement learning is the standard MDP process. MDP can be defined as the four-tuple (S,A,R,P), where S is the state space, A is the action space, R is the reward generated by the environment after performing the action, and P is the state transition function of MDP [27]. The four parameters of MDP are related. Their relationship can be seen as a short sequence $S_{t}\to A_{t}\to R_{t}\to S_{t+1}$, and the state transition function P can be represented as $P_{ss^{\prime }}\left(a\right)=P[S_{t+1}=s’|A_{t}=a,\;S_{t}=s]$.

If the parameters in MDP are known, dynamic programming can be used to calculate the best strategy. If not, the optimal strategy can be obtained through repeated interaction with the environment. In this paper, the Q-learning algorithm in reinforcement learning is used to obtain the optimal selection strategy during the SCF decoding. The advantage of the Q-learning algorithm is that the function max is used for calculation, so the optimal strategy can be quickly found in a simple environment.

The Q-learning is a value-based algorithm in reinforcement learning. There is a Q-table usually denoted by Q(s,a) in this algorithm, where Q(s,a) is the expectation of the reward obtained by the agent taking the action a at the state s [28]. Therefore, the Q-learning algorithm is that S and A form a Q-table to store the value Q(s,a), and then select the action which can obtain the maximum reward value according to the Q-value. In the training of reinforcement learning, an ε-greedy algorithm is often used to explore the state space and action space. When a state transition is determined, Q(s,a) can be updated by Equation (4).

(4) $$Q\left(s,a\right)\leftarrow Q\left(s,a\right)+\alpha \left[r+\gamma {max}_{a^{\prime }}\;Q\left(s^{\prime },a^{\prime }\right)-Q\left(s,a\right)\right],$$

where α is the learning rate and γ is the discounting factor. This formula is the theoretical basis for the Q-learning algorithm.

In the proposed QLSCF algorithm, the selection of candidate bits is offline trained. Once the learning has been completed, the candidate bits obtained from Q-Table are already sorted. The decoding delay caused by the metric ordering can be removed during the decoding process. Therefore, the proposed QLSCF algorithm can reduce the decoding complexity and the decoding delay.

## 3. The Q-Learning-Assisted Successive Cancellation Flip Decoding Algorithm

In this section, we propose a new algorithm which uses the Q-learning-assisted SCF decoding to reduce decoding delay. In order to realize this algorithm, we give the model design of the Q-learning-assisted SCF decoding algorithm.

### 3.1. The Model and Training of the Q-Learning Algorithm

The Q-learning algorithm can be used in the SCF decoding, because the selection procedure of candidate bits is modeled as the MDP process. Selection of candidate bits can be seen as a decision process in general. For the SCF decoding, the decision of the Q-learning is how to select one bit from candidate bits to make the decoded codeword correct.

**Figure 1:**
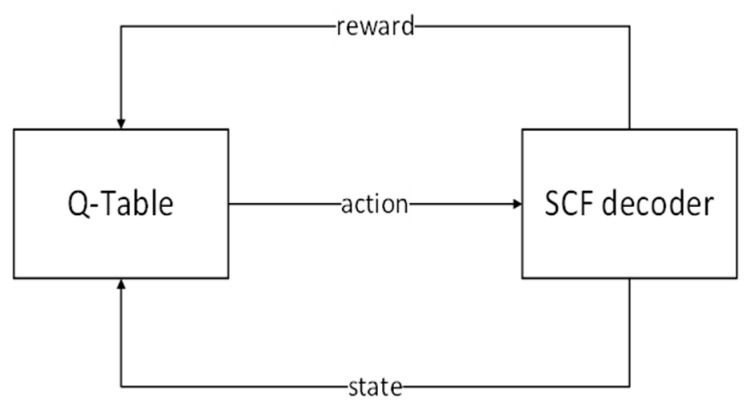
The structure of Q-learning model.

In the above model, the state corresponds to SNR during the decoding, and the action of the state transition corresponds to a candidate bit. The reward function is determined by whether the decoded codeword is correct. The candidate bits set can be obtained from the Q-table. The QLSCF decoding algorithm is modeled by a single-step MDP with a Q-learning algorithm, as shown in Figure 1. The state, action, reward and exploration strategy are detailed as follows.

- The state space S

Discrete SNR values are obtained by systematic sampling method from continuous SNR, ranging between 0.5 dB and 2.5 dB. Nine sampled values of SNR are used as states in state space  S.

- The action space A

**Figure 2:**
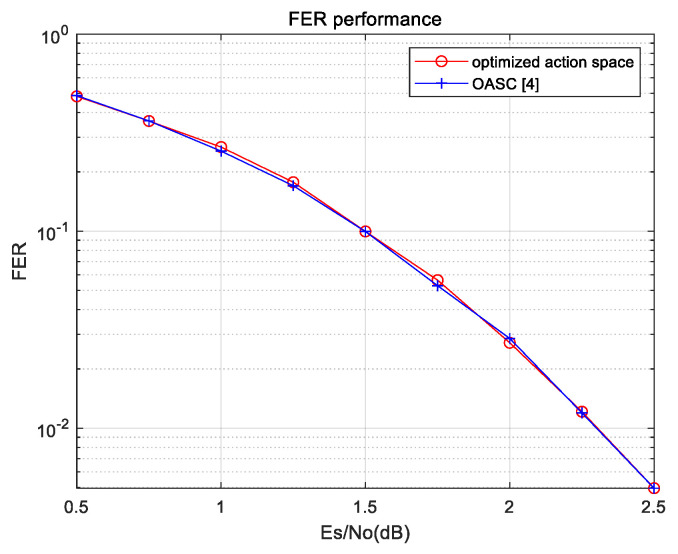
Performance comparison of action space.

We assume that the action space A is an index set, in which action a means that the a-th bit needs to be flipped. Since there are K possible choices, initial action space A is {1, 2, 3, …, K}. However, according to the theory of polar codes, the error probability of each information bit is different, so some indexes of bits are redundant in action space A. Only part of the information indexes is selected as the action space. Therefore, we design an algorithm to obtain the optimized action space, as shown in Algorithm 1. When the code length is 256, the size of initial action space is 128 and the size of the optimized action space is 50. The size of the optimized action space is only half the size of the initial action space. For short polar codes, the frame error rate (FER) of the SC decoding algorithm is greater than 0.5 when SNR is low. Therefore, the decoding codeword has a high error probability. The initial SC decoding is considered as an action in the action space A, which is denoted as $a_{F}$. When SNR is low, the flipping actions may be preferentially performed due to higher FER. Conversely, action $a_{F}$ is preferentially performed when SNR is high. Hence, the agent can decide which action to be preferentially performed through interaction with the environment. In Algorithm 1, θ is an empirical parameter in the optimized algorithm of action space, which has different values for different code lengths. We use this algorithm to effectively reduce the size of the action space A and further reduce the training complexity. The function $SCdecoder\left(y_{1}^{N},\;K,s,\;FEbit\right)$ is a traditional SC decoder used to output the index of the first error bit and the decoded codeword. Then, we also verify the completeness of the optimized action space, as shown in Figure 2. Without considering the decoding delay, the action space is enumerated to participate in bit-flipping decoding, and its decoding performance is compared with the performance lower bound for the one-bit SCF decoding algorithm. The lower bound of the FER performance for the one-bit SCF decoding algorithm is calculated using an Oracle-assisted SC (OASC) decoding algorithm proposed in [4]. The results show that the FER performance of the action space is the same as that of the OASC decoding algorithm. This shows that the size of the action space is reduced without loss of performance. Therefore, the proposed action space is complete.
**Algorithm 1** Optimized design of action space**Input:**The number of trials: T
Information bits length: K
Discrete SNRs: snr
**Output:** The action space: A
**Initialization:**A←0; frame←1; **For**s**in**snr: $Space_{1}^{K}\leftarrow 0$; **While**frame < T: Updating the received LLR: $y_{1}^{N}$; FEbit←Null; //The FEbit is the first error bit index in SC decoding. $\left[{\hat{u}}_{1}^{N},\;FEbit\right]\leftarrow SCdecoder\left(y_{1}^{N},\;K,s,\;FEbit\right)$; **If**
FEbit
**is**
Null: Continue; **else**: Space[FEbit]+=1; frame+=1; **End if** **End while** Space=Space/T; **For**
i
**in**
K: If Space[i]>θ: Move i into A; **End if** **End for** **End for**

- The reward R

Reinforcement learning needs the reward to reflect the effect of action execution. The simple reward function returns to “1” when the flipping decoding is successful, and if the flipping decoding fails, the reward function returns to “−1”. This would imply that an optimal policy is maximum decoding accuracy. This reward function cannot reflect the priority of the candidate bits, because every action may make flipping decoding successful. We introduce the LLR into the reward function and $\left|L_{a}\right|$ is the absolute value of the LLR at action a, as shown in Equation (5). The reward R consists of two parts. One is the feedback of the decoding result. If the action execution makes the decoding codeword correct, the agent will obtain a positive reward “+1”, otherwise a negative reward “−1”. The other is the absolute value of LLR. Since every action may make decoding codeword correct, the reward R of the proposed model should reflect the priority of candidate bits. LLR is added into the reward R, and the absolute value of LLR at action a is denoted as $\left|L_{a}\right|$. The smaller $\left|L_{a}\right|$ is, the higher the error probability of the a-th bit is. Therefore, the reward R can reflect the effect of the action execution, and the Q(s,a) obtained by using this reward R can reflect the priority of the action execution.

(5) $$R=\{\begin{array}{c} -\left|L_{a}\right|+1,\;\;if\;get\;the\;correct\;codeword\;\;\;\;\\ -\left|L_{a}\right|-1,\;\;if\;get\;the\;incorrect\;codeword \end{array},$$

- The exploration strategy

Since each action may make the bit-flipping decoding obtain the correct decoded codeword, we hope that the agent can explore full action space in the early stage of learning. Therefore, the ε-greedy algorithm is modified. An ε(t)-greedy strategy is used as the exploration strategy, where t is the number of training and β represents the decay rate of ε(t), as shown in Equation (6). In the training process of the proposed model, β is 0.004.

(6) $$\varepsilon \left(t\right)=\{\begin{array}{c} 0.5-\beta t,\\ 0.1, \end{array}\begin{array}{c} \;\;\;0<t<\frac{0.4}{\beta }\\ \;\;\;t\geq \frac{0.4}{\beta } \end{array},$$

The Q-learning algorithm for the QLSCF decoding algorithm is described in Algorithm 2, as well as learning parameters being listed in Table 1. The training data is obtained by real-time interacting with the environment during the training process. In the environment, the agent chooses an action to perform SCF decoding in current state and obtains a reward. The process of real-time interaction with the decoding environment can directly reflect the reward of the decision made by the agent. The value of the discounting factor is between 0 and 1. The discounting factor determines the impact of the long-term state on the agent. A larger discounting factor can make the agent tend to learn more far-sighted decision strategy, and a smaller discounting factor can make the agent tend to learn more radical decision strategy. Therefore, the value of the discounting factor is 0.9 in the reinforcement learning algorithm. The value of the learning rate is between 0 and 1. A large learning rate may cause the non-convergent learning results, and a small learning rate may cause a slow convergence rate. Therefore, we made a trade-off to select the value of the learning rate. In Algorithm 2, we set a maximum number of episodes to 1000 and each episode requires multiple Q-value updates.
**Algorithm 2** Q-learning algorithm for the QLSCF algorithm**While**episode < number: Initial observation s; **While**
frame < 10000: Initial information of polar codes: $y_{1}^{N},\;L_{1}^{N}$; **If**
episode < 0.1×number: $a\leftarrow choose\_action\left(s,\;\varepsilon_{l}\right)$; **Else:** $a\leftarrow choose\_action\left(s,\;\varepsilon_{s}\right)$; **End if** $\left(s^{\prime },r\right)\leftarrow env\left(a,\;y_{1}^{N},\;L_{1}^{N},\;s\right)$; $Q\left(s,a\right)\leftarrow Q\left(s,a\right)+\alpha \left[r+\gamma {max}_{a^{\prime }}\;Q\left(s^{\prime },a^{\prime }\right)-Q\left(s,a\right)\right]$; s=s’; frame+=1; **End while** **End while** // The env function **Function**
$\left(s^{\prime },\;r\right)\leftarrow env\left(a,\;y_{1}^{N},\;L_{1}^{N},\;s\right)$: $s^{\prime }\leftarrow getstate\left(s,a\right)$; $flag\leftarrow polardecoder\left(a,\;y_{1}^{N},\;L_{1}^{N},\;s\;\right)$; **If**
flag=1: $r=-\left|L_{a}\right|+1$; **Else:** $r=-\left|L_{a}\right|-1$; **End if** **End function**

The processed Q-table is obtained by sorting the Q-values. In the processed Q-table, each row corresponds to an SNR value. The elements in each row represent actions, in which the actions are sorted according to the Q-value from largest to smallest. This process of the processed Q-table does not participate in the SCF decoding. Therefore, this process does not increase the decoding delay of the proposed QLSCF algorithm.

The detailed description of the proposed functions in Algorithm 2 is as follows.

1. choose_action(s, ε): It is to select the action a at the state s. The function has a probability of ε to randomly select the action a, and a probability of 1−ε to use $\arg max_{a}Q\left(s,a\right)$ to select the action a.
2. $env\left(a,\;y_{1}^{N},\;L_{1}^{N},\;s\right)$: It represents the environment of the Q-learning algorithm. The function can be used to generate the state s’ and the reward r. The specific details of the function are described in Algorithm 2.
3. getstate(s,a): It is used to get the next state s’.
4. $polardecoder\left(a,\;y_{1}^{N},\;L_{1}^{N},\;s\right)$: A decoder of polar codes has additional functions. Depending on the input action a, the decoder performs different decoding. If action a is NF, the decoder performs the traditional SC decoding. If action a is the index of the candidate bits, the decoder performs an additional decoding attempt to decode the codeword with flipping a bit. Finally, the decoder outputs flag used to judge whether the decoded codeword is correct.

### 3.2. Q-Learning-Assisted Successive Cancellation Flip Decoding Algorithm

The QLSCF algorithm uses the reinforcement learning algorithm to select the candidate bits. In the SCF algorithm, the selection of the candidate bits is a decision process. Therefore, an optimal selection strategy needs to be obtained. Reinforcement learning technology can solve decision problems in complex environment and obtain optimal selection strategy through a large amount of learning. The selection of the candidate bits can be seen as the MDP process in the complex environment, so Q-learning is used to obtain an optimal selection strategy. For the conventional SCF algorithms, the values of LLR and the set of candidate bits are obtained after the SC decoding, and the values of LLR are sorted to obtain the order of the candidate bits. For the proposed QLSCF algorithm, the order of candidate bits is already obtained in the reinforcement learning. Hence, the proposed QLSCF algorithm only needs to use the sorted set of candidate bits during the decoding process, and the additional sorting process is not needed.

**Figure 3:**
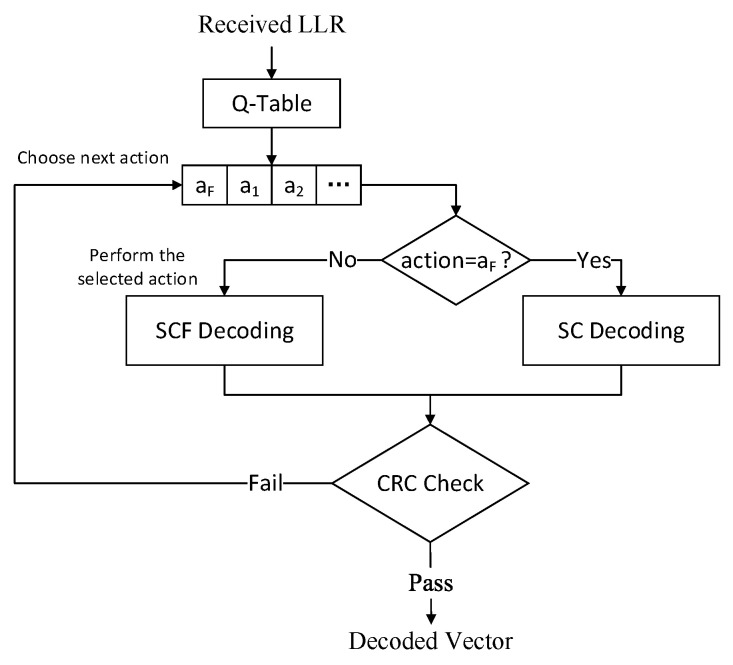
Decoding process of the QLSCF decoding algorithm.

The decoding process of the proposed QLSCF decoding algorithm is shown in Figure 3 and the QLSCF algorithm is shown in Algorithm 3. The candidate bits obtained from Q-Table have been already sorted. In addition, the SC decoding is considered as a special action $a_{F}$, which means to perform the SC decoding. The conventional SCF algorithm firstly performs the SC decoding to obtain the decoded codeword; if the decoded codeword does not pass CRC check, the conventional SCF algorithm will perform the additional bit-flipping decoding. In contrast, the QLSCF algorithm does not have this process. We add action $a_{F}$ into the action space in the Q-learning model. After that, actions are sequentially performed according to the priority of actions after training. Bit-flipping decoding may be firstly performed to reduce the decoding delay when SNR is low.
**Algorithm 3** Q-learning-assisted Successive Cancellation Flip Decoding Algorithm**Input:**The received LLR: $y_{1}^{N}$
Information bits length: K
SNR: snr
The processed Q-table: Q
**Output:** The decoded vector: ${\hat{u}}_{1}^{N}$
**Initialization:**$Q_{flip}\leftarrow getflipset\left(snr,\;Q\right)$; ${\hat{u}}_{1}^{N}\leftarrow QLSCF\left(y_{1}^{N},\;K,\;Q_{flip}\right)$; **Return**${\hat{u}}_{1}^{N}$; // The QLSCF function **Function**${\hat{u}}_{1}^{N}\leftarrow QLSCF\left(y_{1}^{N},\;K,\;Q_{flip}\right)$: **For**i←1**to**$\left|Q_{flip}\right|$: $action\leftarrow Q_{flip}\left(i\right)$; ${\hat{u}}_{1}^{N}\leftarrow SCFdecoder\left(y_{1}^{N},\;K,\;action\right)$; **If**$CRC\;check\left({\hat{u}}_{1}^{N}\right)$ successes: break; **End if** **End for** **Return**
${\hat{u}}_{1}^{N}$; **End function**

The detailed description of the proposed functions in Algorithm 3 is as follows.

1. getflipset(snr, Q): It is to extract the set of candidate bits. Q is the processed Q-table. In the processed Q-table, the actions corresponding to each state are sorted in a descending order of Q-value. As a result, this function outputs the sorted sequence of actions for state snr in the processed Q-table.
2. $SCFdecoder\left(y_{1}^{N},\;K,\;action\right)$: A decoder of polar codes has additional functions. The decoder performs different decoding methods for the input action. If action is NF, the decoder performs the traditional SC decoding. If action is the index of the candidate bits, the decoder performs additional decoding attempt to decode the codeword with flipping a bit. Finally, the decoder outputs the decoded codeword.

## 4. Experiment and Analysis

In this Section, the proposed QLSCF decoding algorithm is compared with other decoding algorithms in terms of the FER performance and the decoding delay. The experimental results validate the advantage of the proposed QLSCF algorithm. All decoding algorithms are simulated with a 0.5 code rate. The maximum number of flips is equal to the size of the critical set. Error detection performance of CRC is also considered. For different decoding algorithms using the same CRC length, their error detection probabilities of CRC are the same. The longer CRC length is, the higher the error detection probability is. However, the long CRC length has a big impact on code rate of polar codes. Therefore, we make a tradeoff between the error detection probability and code rate. The CRC length for each decoding algorithm is set to 16. In the training process of reinforcement learning, the transmitted codeword is known. The binary phase shift keying (BPSK) modulation and the additive white Gaussian noise (AWGN) channel are used in this experiment.

### 4.1. The FER Performance Analysis of the QLSCF Decoding Algorithm

**Figure 4:**
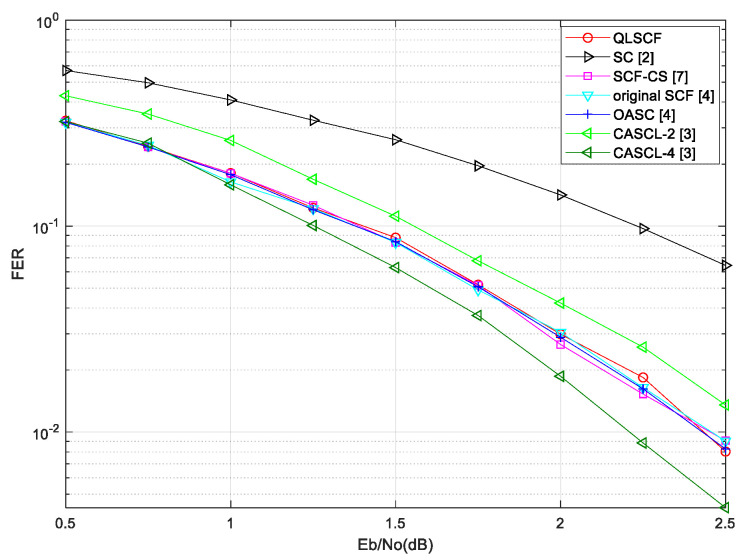
The FER comparison for different decoding algorithms with a code length of 128.

**Figure 5:**
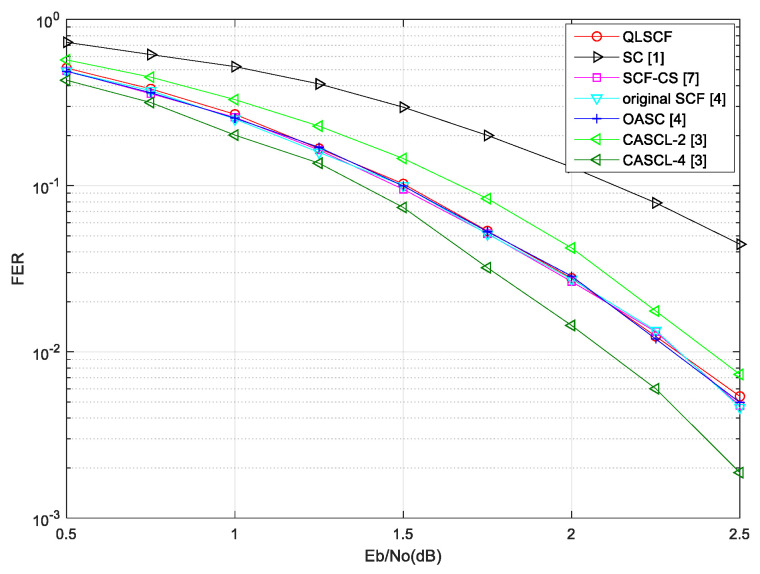
The FER comparison for different decoding algorithms with a code length of 256.

The proposed QLSCF decoding algorithm is compared with traditional decoding algorithms, including the SC decoding algorithm [1], the OASC decoding algorithm [4], the original SCF decoding algorithm [4], the SCF-CS decoding algorithm [7] and the CASCL decoding algorithm with list l=2 and l=4 [3]. The CASCL decoding algorithm with l=2 is denoted as CASCL-2, so does CASCL-4. The FER performance comparisons and the average number of additional decoding attempts are given. The FER performance of the proposed QLSCF decoding algorithm is compared with that of other decoding algorithms in Figure 4 and Figure 5.

Figure 4 shows the FER performance of the proposed QLSCF algorithm with code length of 128 and code rate of 0.5. In Figure 4, the FER performance of the QLSCF algorithm is better than that of both the CASCL-2 and the SC decoding algorithm, and it is almost consistent with the performance of both the OASC decoding and the SCF-CS decoding algorithm. Furthermore, Figure 5 illustrates the FER performance of the proposed QLSCF algorithm and the traditional algorithms with code length of 256 and code rate of 0.5 under the same channel and modulation. In Figure 5, the simulation result shows that the performance of the proposed QLSCF algorithm is better than that of both the CASCL-2 Algorithm and the SC Algorithm. The FER performance of the proposed QLSCF algorithm is less than that of the CASCL-4 decoding algorithm in Figure 4 and Figure 5. However, the CASCL decoding algorithm increases the computational complexity and storage space in exchange for the improvement of decoding performance. In general, the proposed QLSCF Algorithm does not have performance loss compared with both the OASC decoding and the SCF-CS decoding Algorithm.

The comparison of the candidate bits set for different algorithms is shown in Table 2. The candidate bit set of original SCF algorithm contains all information bits. In the simulation, the critical set used by SCF-CS decoding algorithm is obtained at 1.5 dB. The proposed action space includes error positions under multiple SNRs. Therefore, the size of the proposed action space is larger than that of the critical set. The size of the proposed action space is less than that of the bit-flipping set in the original SCF decoding algorithm.

**Figure 6:**
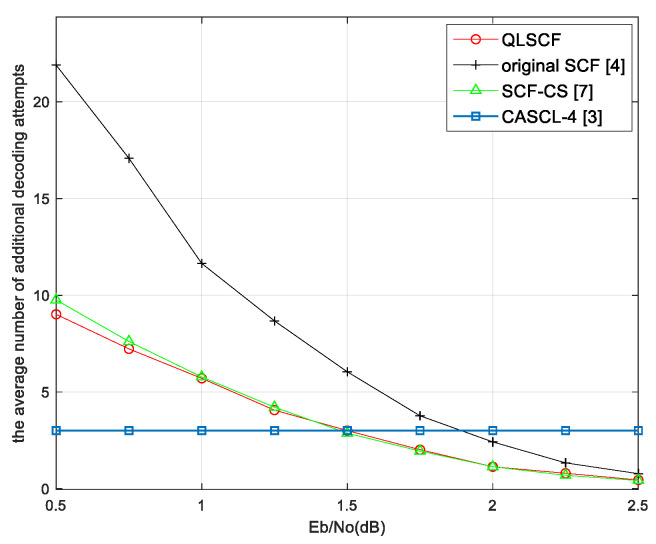
The average number of additional decoding attempts with 128 code length.

**Figure 7:**
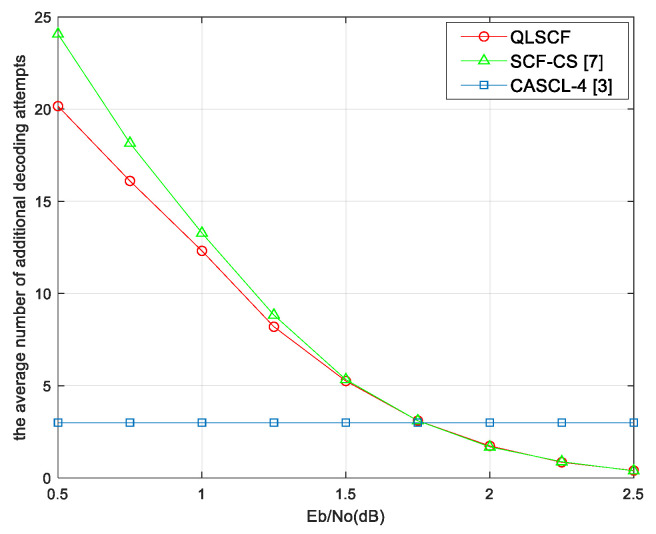
The average number of additional decoding attempts with 256 code length.

In order to analyze the decoding complexity, we count the average number of additional decoding attempts for different algorithms, which are shown in Figure 6 and Figure 7. It can be seen from the results that the average additional decoding attempts of all bit-flipping decoding algorithms decrease as SNR increases and approach 0 at high SNR.

When SNR is less than 1.25 dB, the average additional decoding attempts of the proposed QLSCF algorithm are less than that of both the original SCF and the SCF-CS decoding algorithm in Figure 6. It can be seen in Figure 7 that the average additional decoding attempts of the QLSCF algorithm are reduced compared with the SCF-CS decoding algorithm when SNR is less than 1.5 dB. At high SNR, the average additional decoding attempts of the proposed algorithm are the same as that of the SCF-CS decoding algorithm in Figure 6 and Figure 7. When SNR is high, the complexity of the proposed QLSCF decoding algorithm is lower than that of the CASCL-4 decoding algorithm in Figure 6 and Figure 7. In general, the decoding complexity of the proposed QLSCF algorithm is lower than that of other algorithms when SNR is low.

### 4.2. Decoding Delay Analysis of the QLSCF Decoding Algorithm

The additional decoding attempts and the acquisition of the priority for the candidate bits cause an increase of decoding delay in the SCF decoding. In this subsection, the delay caused by sorting candidate bits are analyzed, so does the overall decoding delay. The decoding delay is affected by the number of time-steps during decoding process [29]. The greater the number of time-steps is, the greater the decoding delay is. The traditional SCF decoding algorithms contain the delay caused by the initial SC decoding, the delay caused by the sorting and the delay caused by the additional decoding attempts. 

For the delay caused by the sorting, the absolute values of the LLR for information bits need to be sorted in the original SCF algorithm, so the decoding delay caused by the sorting is considered as $Klog_{2}K$. In the SCF-CS decoding algorithm, since only |CS| values need to be sorted, t, the decoding delay caused by the sorting, is considered as $\left|CS\right|log_{2}\left|CS\right|$, where CS is the critical set and |CS| is the length of the critical set. In the proposed QLSCF decoding algorithm, the selection of candidate bits is learned by the Q-learning algorithm and the processed Q-table is directly used for the QLSCF decoding. Therefore, the proposed QLSCF decoding algorithm does not need to sort the values of the LLR, and the decoding delay caused by the sorting is considered as 0. Denote the decoding delay caused by the sorting by $T_{sort}$.

The comparisons of decoding delay caused by sorting are shown in Table 3. The proposed QLSCF decoding algorithm has the least decoding delay caused by sorting.

For the overall decoding delay, the delay caused by the initial SC decoding and the delay caused by additional decoding attempts need to be considered. The number of decoding time-steps caused by the initial SC decoding process is equal to 2N−2 [29]. Additional decoding attempts only need to start decoding after the index of a candidate bit, so each attempt requires $\frac{N-I}{N}\left(2N-2\right)$ time-steps to decode a codeword, where I is the index of a candidate bit. Denote the number of decoding time-steps caused by additional decoding attempts by $T_{att}$. The general form of $T_{att}$ is Equation (7).

(7) $$T_{att}=\frac{1}{M}\sum_{i=1}^{M}\sum_{j=1}^{m_{i}}\frac{N-I_{ij}}{N}\left(2N-2\right),$$

where M is the number of information frames, $m_{i}$ is the number of additional decoding attempts for the i-th information frame, and $I_{ij}$ is the index of the candidate bit for the j-th attempt of the i-th information frame.

In summary, the number of total decoding time-steps is equal to $T=2N-2+T_{att}$. The comparison of the total decoding delay is shown in Table 4.

**Figure 8:**
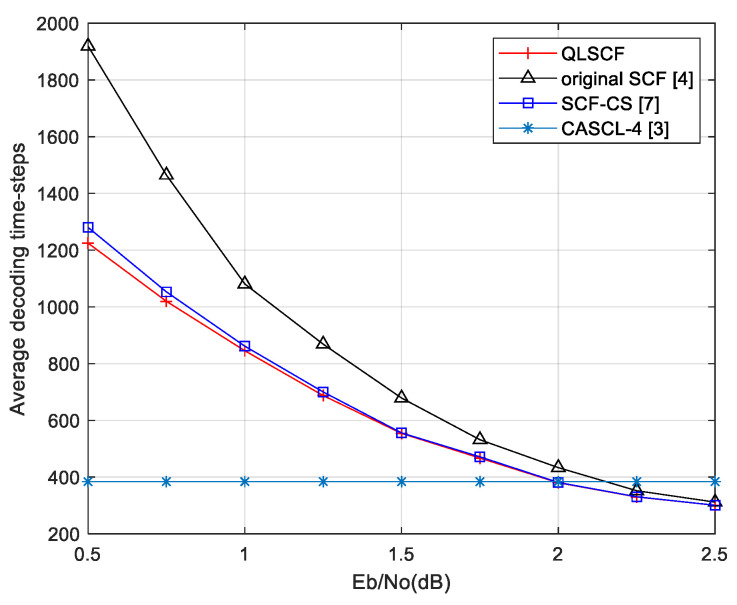
Average decoding time-steps comparison for different decoding algorithms with 128 code length.

**Figure 9:**
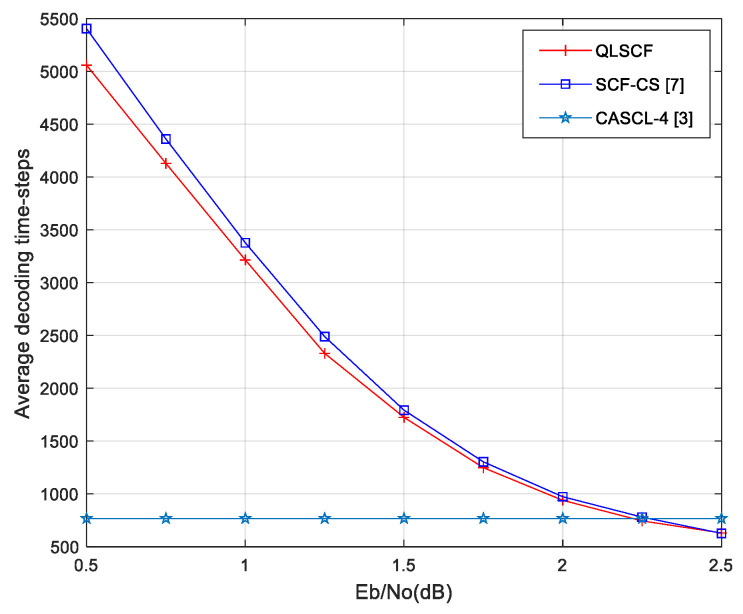
Average decoding time-steps comparison for different decoding algorithms with 256 code length.

The number of additional decoding attempts is different for different decoding algorithms. In order to intuitively illustrate the comparisons of the QLSCF decoding, the original SCF decoding and the SCF-CS decoding algorithm, the total decoding delay is obtained by counting the average decoding time-steps. The decoding time-steps of the proposed QLSCF algorithm are compared with that of other algorithms in Figure 8 and Figure 9.

At low SNR (less than 1.5 dB), the average decoding delay of the proposed QLSCF algorithm is less than that of both the original SCF and the SCF-CS decoding algorithm in Figure 8. When SNR is greater than 2 dB, the decoding delay of the proposed QLSCF decoding algorithm is lower than that of the CASCL-4 decoding algorithm in Figure 8. The decoding delay of the proposed QLSCF algorithm is reduced compared with the SCF-CS decoding algorithm when SNR is 1.25 dB.

In Figure 9, the decoding delay of the proposed QLSCF algorithm is less than that of the SCF-CS decoding algorithm at low SNR (less than 2 dB). When SNR is greater than 2.25 dB, the decoding delay of the proposed QLSCF decoding algorithm is lower than that of the CASCL-4 decoding algorithm. The decoding delay of the proposed QLSCF algorithm is reduced compared with the SCF-CS decoding algorithm when SNR is 1.75 dB.

At high SNR, the decoding delay of the proposed QLSCF algorithm is the same as that of the SCF-CS decoding algorithm and less than that of the original SCF decoding algorithm. The above results imply that we can apply the Q-learning algorithm to an SCF decoding algorithm to reduce decoding delay without loss of performance.

The SCF decoding algorithm based on LSTM network [26] uses reinforcement learning in the decoding process. However, the decoding delay caused by LSTM network is not given. When the proposed QLSCF algorithm is compared with this algorithm, the average decoding time per frame can be used to evaluate the decoding delay under the same hardware conditions.

## 5. Conclusions

A Q-learning-assisted successive cancellation flip decoding algorithm has been presented, which uses the reinforcement learning assisted SCF decoding to reduce decoding delay. A reinforcement learning model for the SC bit-flipping decoding is proposed, and the SCF decoding algorithm is executed by using reinforcement learning algorithm to select candidate bits. The proposed QLSCF algorithm selects the candidate bits to decode the codeword through the learned Q-table rather than the metric sorting. For other code lengths, the proposed algorithm is also applicable. It only needs to construct the action space under the corresponding code length to learn in reinforcement learning model. The proposed QLSCF decoding algorithm reduces decoding delay without loss of performance at low SNR. Future research directions involve reinforcement learning model of the multi-bit SCF decoding and further optimization of the proposed algorithm.

## Figures and Tables

**Table 1:** Q-Learning Algorithm Hyperparameters.

Hyperparameters	Values
Learning rate	0.01
Discounting factor	0.9
ε-greedy	ε(t)
Reward	$-\left|L_{a}\right|+1$ or $-\left|L_{a}\right|-1$

**Table 2:** The comparison of the candidate bits set for different algorithms with 256 code length.

Algorithm	The Size of Candidate Bits Set
SCF-CS algorithm [7]	39
original SCF algorithm [4]	128
QLSCF algorithm	51

**Table 3:** Decoding Delay Caused by Sorting During the Decoding Process.

Algorithm	$T_{sort}$
original SCF algorithm [4]	$Klog_{2}K$
SCF-CS algorithm [7]	$\left|CS\right|log_{2}\left|CS\right|$
QLSCF algorithm	*0*

**Table 4:** Total Decoding Delay During the Decoding Process.

Algorithm	T
original SCF algorithm [4]	$2N-2+\frac{1}{M}\overset{M}{\underset{i=1}{\sum }}\overset{m_{i}}{\underset{j=1}{\sum }}\frac{N-I_{ij}}{N}\left(2N-2\right)$
SCF-CS algorithm [7]	$2N-2+\frac{1}{M}\overset{M}{\underset{i=1}{\sum }}\overset{m_{i}}{\underset{j=1}{\sum }}\frac{N-I_{ij}}{N}\left(2N-2\right)$
QLSCF algorithm	$\frac{1}{M}\overset{M}{\underset{i=1}{\sum }}\overset{m_{i}}{\underset{j=1}{\sum }}\frac{N-I_{ij}}{N}\left(2N-2\right)$

## Data Availability

Not applicable.
